# Speaking the same language – a scoping review to identify the terminology associated with social prescribing

**DOI:** 10.1017/S1463423623000567

**Published:** 2023-11-28

**Authors:** Simon Newstead, Megan Elliott, Dawn Cavanagh, Sion Tetlow, Carolyn Wallace

**Affiliations:** 1 Life Sciences and Education, University of South Wales, Treforest, UK; 2 Wales School for Social Prescribing Research (WSSPR), UK; 3 Local Public Health Team, Cwm Taf Morgannwg University Health Board, Cardiff, UK; 4 Welsh Institute for Health and Social Care University of South Wales, Treforest, UK

**Keywords:** community connection, glossary of terms, scoping review, social prescribing, terminology

## Abstract

**Aim:**

To identify the social prescribing-related terminology within the peer-reviewed literature of the UK and the grey literature from Wales.

**Background:**

Social prescribing has seen a period of development that has been accompanied by a proliferation of related terminology and a lack of standardisation in the manner in which it is employed. This creates barriers to engagement and impairs communication, both between professionals and members of the public. The Wales School for Social Prescribing Research and Public Health Wales committed to the development of a glossary of terms for social prescribing, to facilitate the clarification and standardisation of the associated terminology. Here, we describe the first step in that process.

**Method:**

A scoping review of the peer-reviewed UK literature and Welsh grey literature was conducted. The titles and abstracts of 46,242 documents and the full text of 738 documents were screened. Data were charted from 205 documents. Data capture included terminology, the location within the UK of the research or intervention described in the article, and the perspective from which the article was authored. A general inductive approach was used to categorise the terms by theme.

**Findings:**

This research serves to highlight the breadth and diversity of the terminology associated with social prescribing. Results demonstrate aspects of shared commonality and clear distinction between the terminology from the two literature sources. The greatest contributions of terms were from articles that examined research and/or interventions in England and that were authored from the perspective of health or health and social care. The research indicates that nation- and sector-specific terms may not be adequately represented in the literature at large. Looking forward, it will be important to ensure that social prescribing terminology within the UK literature is culturally relevant and accurately reflects the terminology used by the workforce who encounter and deliver social prescribing.

## Introduction

Social prescribing, which may also be referred to by other titles such as connector schemes (Tierney *et al.*, 2020), community referral (Husk *et al.*, 2016; All Ireland Social Prescribing Network, 2021), and care navigation (Pesut *et al.*, 2017; NHS Inform, 2022), describes a pathway that uses a person-centred approach to lift up individuals through engagement with a broad range of different community-based activities and services (Kimberlee, 2015; SCIE, 2020). Examples of such community-based activities and services include art-based activities, social cafes, tenancy support services, and swimming, walking, and gardening groups. Although the term ‘social prescribing’ might imply an action of authoritative direction or be accompanied by perceptions of paternalism (Brown *et al.*, 2021), the term is a bit of a misnomer as the ethos that underpins the practice is one of empowerment (Kimberlee, 2015; SCIE, 2020).

The social prescribing pathway begins with the act of referral. The simplest form of social prescribing is where a professional simply directs the individual to a potentially useful service. A more holistic form, which better represents the practice of social prescribing, involves referral to a social prescribing practitioner (Kimberlee, 2015; SCIE, 2020). There are multiple avenues of referral into the social prescribing pathway, including via healthcare, social care, the third sector, and by the individual in question referring themselves to a social prescribing practitioner (Welsh Government, 2022). The social prescribing practitioner will support the individual to identify what their needs are and what is important to them. They will then help them to produce an action plan, identify suitable activities and support in the community, and then support them to engage with these community assets (Thomas *et al.*, 2021). Through engagement with this process, social prescribing can help individuals address concerns such as isolation, weight or health concerns, or financial worries, thereby providing them with greater control over their circumstances and well-being (Drinkwater *et al.*, 2019; Wood *et al.*, 2021). Individuals can access the same community-based activities and services themselves directly, outside of the social prescribing pathway, but this would not be considered to be social prescribing.

While the elements that constitute social prescribing are relatively consistent across the nations of the UK, each has its own definition (see All Ireland Social Prescribing Network, 2021; NHS England, 2023; Rees *et al.*, 2019; NHS Inform, 2022). Within Wales, social prescribing is currently defined as ‘connecting citizens to community support to better manage their health and well-being’ (Rees *et al.*, 2019). Wales has developed a cross-sectional model of social prescribing that is integrated with existing community and statutory services (Public Health Wales, 2018; Wallace *et al.*, 2021) and aligns with relevant policies, such as the Social Services and Wellbeing (Wales) Act (Welsh Government, 2014) and the Wellbeing and Future Generations (Wales) Act (Welsh Government, 2015). This model has moved away from the medical model of care, instead focussing on holistic and person-centred methods (Pringle & Jesurasa, 2022) to help empower individuals to recognise their own needs and strengths and to connect with their communities for support with their health and well-being (Welsh Government, 2022).

Social prescribing has seen a period of proliferation and development over the last decade (Bertotti *et al.*, 2018; Morse *et al.*, 2022; Rempel *et al.*, 2017). The pace of this development has led to a lack of standardisation of several aspects of social prescribing, such as associated objectives, job roles, and outcomes, as well as the associated terminology (Rempel *et al.*, 2017; Wallace *et al.*, 2021). For example, social prescribing practitioners may have various titles such as link worker, community connector, well-being advisor, care navigator, or social prescriber (Carnes *et al.*, 2017; Hamilton-West *et al.*, 2019; Tierney *et al.*, 2020; Wallace *et al.*, 2021; Wallace *et al.*, 2019). The diversity and the lack of standardisation of the terminology associated with social prescribing are confusing for professionals and the public alike, impairing effective communication and creating barriers to engagement (Brown *et al.*, 2021; Wallace *et al.*, 2021)

The Wales School for Social Prescribing Research (WSSPR) is a virtual all-Wales school that hosts the Wales Social Prescribing Research Network (WSPRN) and nests within PRIME Centre Wales. THE WSPRN has a diverse membership of over 350 researchers and professionals and supports three communities of practice, which feed out to members of the public and the social prescribing community across Wales (WSSPR, 2022). Through consultation with members of the public, the WSPRN identified a need for a reference tool to provide clarity on the terminology associated with social prescribing. Consequently, WSSPR and Public Health Wales committed to the development of an evidence-based glossary of terms for social prescribing in Wales (Newstead *et al.*, 2023; Wallace *et al.*, 2018).

Here, we report on the first stage of this process, the completion of a scoping review of the social prescribing-related literature, to identify, collate, and categorise the social prescribing-related terminology contained within the peer-reviewed journal articles of the UK and the grey literature of Wales.

## Method

A scoping review was identified as the most appropriate form of literature review for this research, as the aim of the research was to map, report, and discuss the characteristics/concepts within a body of literature (Munn *et al.*, 2018), without assessing the quality of the included studies (Arksey & O’Malley, 2005). The scoping process incorporates a comprehensive search for information, guided by an a priori protocol (Peters *et al.*, 2015), and structured analyses of the literature (Davis *et al.*, 2009; Levac *et al.*, 2010; Peters *et al.*, 2015). This may be an iterative process that requires reflexive engagement to ensure a comprehensive review of the relevant literature (Peters *et al.*, 2015; Peterson *et al.*, 2017).

### Protocol

Our protocol was preregistered with the Open Science Framework: shorturl.at/jNY79. The protocol was based on the scoping review methodological framework proposed by Arksey & O’Malley (2005), which employed a five-stage process (outlined below). It also incorporated the suggestions by Levac *et al.*, (2010) of including a numerical summary and qualitative thematic analysis to help identify the implications of the study findings for policy, practice, or research. As is common with scoping reviews, the process was refined as familiarity and an appreciation of the breadth of material was gained.

### Stage 1: identifying the research question

Our research question was ‘What terminology is associated with social prescribing and the social prescribing pathway?’

### Stage 2: identification of relevant studies

Peer-reviewed literature was identified by searching 11 electronic databases using a comprehensive list of 49 search terms that had been identified as relevant to social prescribing by members of the WSPRN and WSSPR steering group (a list of search terms and databases is available to view at shorturl.at/jNY79). Searches were conducted between 01/05/2021 and 30/06/2021 and were restricted by date (post-1980), language (English only), and location (UK). The location was set as the UK instead of Wales due to the absence of a Wales filter on some of the databases and the known influence of UK peer-reviewed literature language on social prescribing terminology within Wales. The types of documents deemed eligible for inclusion were peer-reviewed articles in academic journals including case studies, editorials, opinion pieces, studies, and experiments.

Welsh social prescribing grey literature was sourced from the Welsh Government, Welsh local authority, third sector, and university websites, identified using the search engine Google as well as recommendations from professionals associated with WSSPR and the social prescribing communities of practice in Wales. Grey literature searches were conducted between 01/03/2022 and 15/04/2022 and again restricted by date (post-1980) and language (English only). The types of documents deemed eligible for inclusion were guidance, reports, working papers, government documents, white papers, and evaluations and specialist magazine articles (e.g., specific to health or social care).

To further increase our information capture, snowball searches were conducted on all items deemed relevant. Items accepted for analysis were searched for additional references relating to our search, which were then subsequently sourced and examined.

### Stage 3: selection of studies/literature

The selection of documentation for analysis and identification of social prescribing-related terminology underwent a two-step eligibility screening process:

Step 1 **Screening:** Titles and abstracts from peer-reviewed literature and the title and overview/foreword from grey literature were screened to determine if they held relevance to the nature of our search, i.e., do they:

- Include one of the search phrases in the context of social prescribing as defined by the involvement of a link worker or navigator who works with people to access local sources of support.
- Allude to the facilitation of an external community/voluntary service by means of an intermediary specialist.

We also included literature that referred to interventions known to occur in social prescribing but that had not fulfilled one of the above conditions in the abstract or foreword. These included: (1) physical activity programmes such as exercise, dance, guided walks, team sports, and cycling, (2). nutrition interventions such as diet clubs, food clubs, weight management, and diet therapy, and (3). psychosocial support such as social support groups, befriending, art-based activities, and green and blue space interventions.

Step 2 **Screening:** Details of all items identified as potentially relevant during stage 1 screening were recorded in a database, including items identified during snowball searches. Step 2 screening involved screening the text of each document for social prescribing-related terminology. This was defined as terminology that was explicit to, or associated with, social prescribing and the social prescribing pathway.

### Stage 4: charting the literature and data

The data from items that were deemed relevant during step 2 screening were input into an Excel database to create a data charting form (by the first author, SN). The data that were collected are described in Table 1. As a quality control measure, 20% of the documents underwent independent step 2 screening and charting (by the team, DC, ME, ST) with periodic collation and consensus meetings to ensure that terms were not being overlooked.

**Table 1.** Information collated in data charting form

**General information**	Document number, title, reference, source (e.g., peer/grey, ProQuest, snowball search)
**Document classification**	Intervention review, literature review, systematic review, scoping review, case study, qualitative study, quantitative study, mixed methods study, interest article/opinion piece, future research, act/plan, strategy, guidance, newsletter, evaluation/report, project overview, presentation
**Contextual information**	**Term**
	**Description**: As included in the article, plus contextual information provided by the article
	**Author perspective:** The overarching perspective from which the article is authored, accounting for the journal, the authors specialty/role, the content, and the context of the article
	**Location:** The nation in which the research/intervention was conducted, or UK if the research/intervention was not specifically related to a devolved nation

### Stage 5: collating, summarising, and reporting the results

The number of documents and terms was summarised by source (peer-reviewed and grey literature), the location of the research or intervention described within them, and the perspective from which the articles were authored. A general inductive approach (Thomas, 2006) was taken to categorise the various themes of the terminology identified from the scoping review; terms were manually coded, and the codes were then grouped to produce common themes. The database of charted data can be viewed at shorturl.at/jNY79. Charting the information from the articles identified in our research allowed us to present a basic numerical analysis of the information collated, as well as a narrative of our findings.

## Results

All searches were examined in their entirety, except for those for the search of PubMed, which returned 2 341 050 items. The search was subsequently restricted to documents, reviews, and systematic reviews, which reduced the number of items to 290 000. We then used a function of the database to sort the remaining terms by relevance and examined the first 5000, as no potentially relevant items were identified after the first 4000 items indicating data saturation (Saunders *et al.*, 2018).

**Figure 1.**
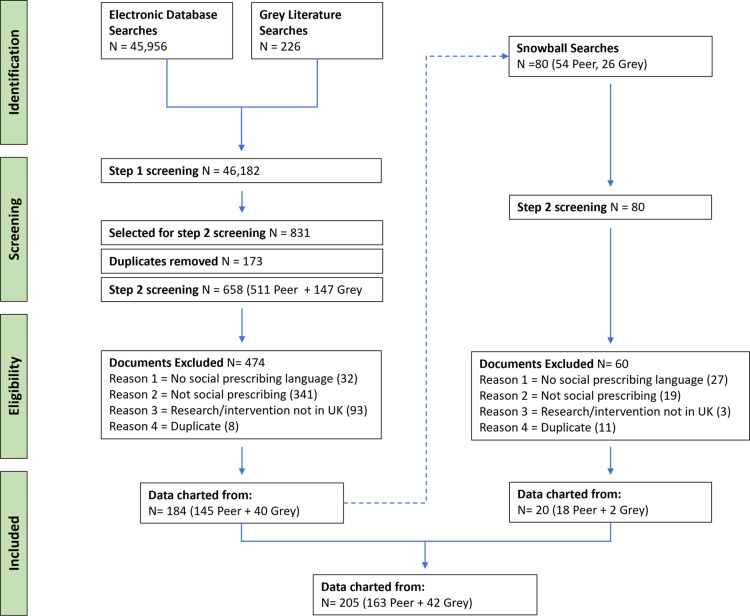
PRISMA diagram of the scoping process.

A total of 817 term data points were initially charted from 163 peer-reviewed documents and 42 grey literature documents. Following the removal of duplicate terms, 373 different terms were identified (see Fig. 1).

### Distribution of the identified terms by source and location

The distribution of terms by source and location of the research/intervention described within them can be viewed in Table 2. Examination of the terms by source showed that 70% of terms (n = 260) occurred solely within the peer-reviewed literature and 20% of terms (n = 75) occurred solely in the grey literature, with a co-occurrence of 10% (n = 38) of the identified terms across both sources. A greater number of peer-reviewed documents were examined in their entirety and a higher number of terms were identified in the peer-reviewed literature than in the grey literature. However, examination of the average concentration of terms per article indicated a higher total concentration of terms per article in the grey literature (2.69 terms/article) than in the peer-reviewed literature (1.83 terms/article).

**Table 2.** Total number of individual terms identified by document source and location

Location	Peer-reviewed documents		Grey literature	
	Documents	Terms	Documents	Terms
England	81	191 (2.35)		
Scotland	11	27 (2.45)		
Northern Ireland	2	6 (3)		
Wales	6	17 (2.83)	42	113 (2.69)
UK	62	122 (1.97)		
Total	163	298 (1.83)	42	113 (2.69)

NB: () shows the average number of terms per document.

Further examination of the terms identified from the peer-reviewed literature, by the location of the research/intervention described within them (Table 3), revealed that approximately half of the terms identified originated from articles with a research perspective based within England, with approximately a further quarter originating from articles with a UK wide research perspective. The largest co-occurrence of terms was found within the literature from the UK and England. Peer-reviewed articles that described research/interventions in the other nations of the UK contributed relatively few terms. Only two terms co-occurred across the literature from all nations: ‘social prescribing’ and ‘link worker’. While a shared commonality in the terminology used by the different nations of the UK is apparent, there is also a clear distinction. Respectively, approximately 60%, 40%, and 33% of the terms used in the peer-reviewed literature of Scotland, Wales, and NI solely occurred in the articles produced by those nations.

**Table 3.** Sole and co-occurrence of individual terms by document source and location

Location of research or intervention	Peer-reviewed documents		Grey literature	
	Sole occurrence	Co-occurrence	Sole occurrence	Co-occurrence with peer-reviewed documents
England	147			10
Scotland	16			1
Northern Ireland (NI)	2			
Wales	7		75	1
UK	77			10
All Nations		2		2
England and Scotland		2		
England and Wales		2		1
England and UK		28		4
England, Scotland, and UK		4		2
England, NI, and UK		1		1
England, Wales, and UK		5		5
Scotland and UK		3		
NI and UK		1		
Wales and UK		1		
Total	298		113	

One-third of the terms identified in the Welsh grey literature co-occurred in the peer-reviewed literature (Table 3). The largest co-occurrences were with articles that described research/interventions in England and across the UK. Only one term ‘time credits’ co-occurred within the grey literature of Wales and the peer-reviewed articles that described research/interventions in Wales. Again, only the terms ‘social prescribing’ and ‘link worker’ occurred across all nations and co-occurred within the grey literature.

### Distribution of the identified terms by source and authored perspective

A total of seven authored perspectives were identified (Table 4). Articles authored from the perspective of psychology were only found within the peer-reviewed literature, and articles authored from the perspective of community and voluntary service organisations (CVSOs) and health, CVSOs, and health and social care were only found within the grey literature. Examination of the terms by source indicated that the largest contribution of terms originated from articles with an authored perspective of health and health and social care, within both the peer-reviewed literature (approximately 2:1 respectively) and grey literature (approximately 1:1 respectively).

**Table 4.** Total number of individual terms identified by document source and authored perspective

Authored perspective	Peer-reviewed documents		Grey literature	
	Documents	Terms	Documents	Terms
CVSOs	6	15 (2.5)	3	8 (2.67)
Health	106	214 (2.02)	10	41 (4.1)
Health and Mental Health (HMH)	15	31 (2.06)	5	25 (5)
Health and Social Care (HSC)	30	94 (3.13)	10	40 (4)
Psychology	6	24(4)		
CVSOs and Health (CVSOs and H)			12	32 (2.67)
CVSOs and HSC			2	5 (2.5)
Total	163	298 (1.83)	42	113 (2.69)

NB: CVSOs = community and voluntary sector organisations, () shows the average number of terms per document.

Further examination of the terms identified from the peer-reviewed literature, by authored perspective (Table 5), revealed that approximately half of the terms identified originated from articles authored solely from the perspective of health. Approximately a further 20% originated from articles authored solely from the perspective of health and social care. The largest co-occurrence of terms (approximately 10%) was from articles authored from a health perspective and a health and social care perspective. The contribution of terms that occurred in articles authored solely or partially from the perspective of CVOSs was competitively small (approximately 5%). No terms co-occurred across the literature from all of the authored perspectives that were identified in the peer-reviewed literature.

**Table 5.** Sole and co-occurrence of individual terms by document source and authored perspective

Authored perspective	Peer-reviewed documents		Grey literature	
	Sole occurrence	Co-occurrence	Sole occurrence	Co-occurrence
CVSOs	9		2	2
Health	158		25	13
Health and Mental Health (H and MH)	16		18	4
Health and Social Care (H and SC)	51		21	16
Psychology	5			
CVSOs and Health (CVSOs and H)			22	
CVSOs and HSC			2	
CVSOs/Health		1		2
Health/HMH		5		
Health/HSC		30		5
Health/Psychology		8		
HMH/Psychology		2		
HSC/Psychology		1		
CVSOs/Health/HSC		2		
Health/HMH/HSC		2		2
Health, HSC/Psychology		2		
Health/HSC/HMH/Psychology		3		
CVSOs/Health/HMH/HSC/Psychology		3		
CVSOs and H/Health				2
CVSOs and H/HSC				3
CVSOs and HSC/HSC				1
HMH/HSC				2
CVSOs and H/HMH/HSC				1
CVSOs and H/Health/HSC				2
CVSOs/CVSOs and H/Health/HMH/HSC				1
CVSOs/CVSOs and H/CVSOs and HSC/Health/HMH/HSC				1
All authored perspectives				1
Total	298		113	

NB: CVSOs = community and voluntary sector organisations, /indicates terms co-occurring in articles from different author perspectives.

Within the grey literature, the largest sole occurrence of terms was in articles authored from the perspective of health (approximately 20%). However, the contribution of terms that solely occurred in articles authored from the perspectives of health and mental health, health and social care, and CVSOs and health was relatively comparable. In general, there was a low co-occurrence of terms across articles authored from joint perspectives, and only the term ‘social prescribing’ co-occurred across the literature from all of the authored perspectives. However, in contrast to the peer-reviewed literature, the contribution of terms that occurred in articles authored solely or partially from the perspective of CVOSs was comparatively high (approximately 46%). The highest rates of co-occurrence with peer-reviewed literature were in articles authored from the perspectives of health, and health and social care.

### Distribution of terms by source and theme

The identified terms were collated into nine themes. Table 6 provides a breakdown of the number of terms found within each theme, by source, along with example terms.

**Table 6.** Sole and co-occurrence of individual terms by document source and authored perspective

Themes	Total	Peer-reviewed documents			Grey literature			Co-occurring terms	
		Total	Sole occurrence	Example	Total	Sole occurrence	Example		Example
Alternative terms for social prescribing	32	30	25	Linking schemes	7	2	Link worker schemes	5	Community referral
				Non-medical referral			social prescribing intervention		Social prescribing
Essential elements of the practice of social prescribing	64	53	41	Asset-based community development	23	11	Active involvement	12	Asset-based approach
				Linkage route			Feedback loop		What matters conversation
Social prescribing practitioner roles	55	43	32	Community links practitioner	23	12	Community coordinator	11	Community connector
				Social prescribing coordinator			Well-being advisor		Social prescriber
Non-social prescribing practitioner roles	16	16	15	Allied Health Professional	1			1	Social prescribing champions
				Managerial Social Prescriber					
Community-based activities and services	92	67	66	Exercise on prescription	25	24	Lifestyle hubs	1	Art-based approaches
				Healing arts			Well-being hubs		
Social prescribing models and approaches	32	26	21	Social prescribing ‘plus’	11	6	Additionality	5	Holistic model
				Strategic link worker-based model			Bottom-up		Community-enhanced social prescribing
Measures of efficacy	18	11	10	Health and well-being outcomes	8	7	Indicators	1	Measures of efficacy
				Well-being star			Social return on investment		
Barriers and enablers to social prescribing	13	8	7	Buddy system	6	5	Clinical barriers	1	Time credits
				Inappropriate referrals			Individual barriers		
Broadly associated with social prescribing	51	43	42	Frequent attenders	9	8	Share learning	1	Patients
				Time banks			Uplift funding		

Several themes may require some explanation to fully appreciate what the themes encompass. The theme ‘essential elements of the practice of social prescribing’ includes terms that are widely described in the literature as being an important part of the role of the social prescribing practitioner and/or the social prescribing pathway, but that do not sit within the other themes. The theme ‘non-social prescribing practitioner roles’ includes terms that relate to the functioning and/or development of social prescribing in general and/or that describe roles within social prescribing that may administer aspects of social prescribing, but for which social prescribing is not their main role. The theme ‘measures of efficacy’ includes terms that are either specific measures of efficacy found within the examined literature or which relate to the process of establishing the efficacy of a service or intervention. The theme ‘broadly associated with social prescribing’ includes terms that do not easily sit within any of the other themes. The terms may be more specific to other services and/or sectors but may be related to, and/or encountered in, the practice of social prescribing more generally.

The theme ‘community-based activities and services’ contained the largest concentration of terms. The themes ‘essential elements of social prescribing’, ‘social prescribing practitioner roles’, and ‘broadly associated with social prescribing’ also contained relatively high concentrations of terms.

Examination of the terms by source indicated that the greatest concentrations of terms that occurred solely within the peer-reviewed literature were for the themes ‘community-based activities and services’, ‘essential elements of social prescribing’, and ‘broadly associated with social prescribing’. The greatest concentrations of terms that occurred solely within the grey literature were for the themes ‘community-based activities and services’, ‘social prescribing practitioner roles’, and ‘essential elements of social prescribing’. The four themes ‘the act of social prescribing’, ‘essential elements of social prescribing’, ‘social prescribing practitioner roles’, and ‘social prescribing models and approaches’ displayed relatively high levels of co-occurrence of terms across both sources. All other themes only had a single co-occurrence between the two literature sources.

## Discussion

Through research and consultation with professional and public involvement members and social prescribing professionals within Wales, WSSPR identified a need for a means to help address the confusion for professionals and the public alike regarding the terminology associated with social prescribing within Wales (Evans *et al.*, 2020; Wallace *et al.*, 2018). To this end, WSSPR and Public Health Wales committed to the development of a glossary of terms for social prescribing, the first step of which was the identification and categorisation of the terminology associated with social prescribing. To the best of our knowledge, the scoping review described in this article is the first research to attempt to identify, collate, and categorise the social prescribing-related terminology found within the peer-reviewed literature of the UK. Terminology from the Welsh grey literature was also captured, and data were compared across both sources. Terms were collated and examined by the location of the research/intervention described within the articles, by the perspective from which the articles were authored and by theme. A total of 377 terms associated with social prescribing were identified. There was a much larger proportion of peer-reviewed literature than Welsh grey literature, and this was accompanied by a larger contribution of terms. There was an overlap of the terminology used in the peer-reviewed literature of the UK literature and that used in the grey literature within Wales. However, the present research also provides several examples of clear division between the two literature sources. Findings and implications are discussed.

The greatest contributions of terms were from peer-reviewed articles that described research/intervention from England or that were UK-wide. Peer-reviewed articles describing research/interventions for the other nations of the UK were comparatively low, which translated into smaller contributions of terms by these nations. One possible explanation for the disparity between the number of social prescribing-related journal articles for the nations of the UK is that the practice of social prescribing in England is more firmly established than for the other nations of the UK. England has already developed a framework for social prescribing practitioners/link workers (NHS England, 2023) and Wales is in the process of developing a national framework for social prescribing (Welsh Government, 2022). Comparatively, social prescribing in Scotland and NI may be considered to be in their infancy. This explanation is supported by the earliest dates of the peer-reviewed articles identified within this scoping review for each nation of the UK (e.g., England: Fox *et al.*, 1997; Scotland: Cawston, 2011; Wales: Courtenay *et al.*, 2017; and NI: Loftus *et al.*, 2017). The results also indicate aspects of both shared commonality and clear distinction in the terminology used by the different nations of the UK in the examined peer-reviewed literature. The co-occurrence of terms across nations likely reflects, at least in part, the general use of certain terms in practice. However, the relatively low contributions of articles from the other nations of the UK make it impossible to establish the degree to which the terminology used to report research/interventions in England has been adopted.

Examination of the peer-reviewed literature by authored perspective showed that the greatest contributions of terms were from articles authored from the perspectives of health and health social care. This predominance of the publication of social prescribing-related literature from a health or health and social care perspective may, in part, be reflective of a systematic bias for publication from these sectors. Research indicates that many CVSOs struggle to evidence their activities in the peer-reviewed literature (Breckell *et al.*, 2010; Ellis & Gregory, 2008; Despard, 2016; Mitchell & Berlan, 2018), due to a number of barriers including a lack of financial resources, expertise and internal capacity, and a mismatch between the requirements of those funding the service and what the CVSOs perceive to be appropriate evaluation goals (Bach-Mortensen & Montgomery, 2018). Examination of the sole and co-occurrence of terms within the Welsh grey literature largely reflects a similar trend for the contribution of terms from articles authored from a health or health and social care perspective. However, the Welsh grey literature also demonstrated a much larger contribution of terms authored solely or partially from the perspective of CVOSs than was present in the peer-reviewed literature, which may be reflective of differences in the social prescribing models between the two nations. Social prescribing in England is heavily embedded in primary care (Frostick & Bertotti, 2019; King’s Fund, 2020). In contrast, social prescribing in Wales has moved away from the medical model of care (Pringle & Jesurasa, 2022) and has developed a cross-sectional model of social prescribing that is integrated with existing community and statutory services (Public Health Wales, 2018; Wallace *et al.*, 2021) and that currently sits more firmly in the third sector (Elliot *et al.*, 2021).

Thematic analysis of the terms identified four themes. The results indicate aspects of shared commonality and of clear distinction between the terminology used in the examined peer-reviewed literature and the Welsh grey literature. The highest contributions of terms in both the peer-reviewed and Welsh grey literature were found in the themes that relate to the central aspects of the social prescribing pathway ‘community-based activities and services’, ‘essential elements of social prescribing’, and ‘social prescribing practitioner roles’. The co-occurrence of terms between the two sources was predominantly constrained to just four themes, the three aforementioned themes and the theme ‘the act of social prescribing’. However, levels of co-occurrence within these themes were also approximately equal to the number of terms that solely occurred within the Welsh grey literature. Each of the other five themes had only a single instance of co-occurrence of terms between the two sources. Surprisingly, Welsh grey literature contributed only a single term to the theme ‘non-social prescribing practitioner roles’. While it is beyond the scope of this research to ascertain, this observed disparity in the concentration of terminology across source and theme may be reflective of several factors: the prevalence and influence of English or UK peer-reviewed literature informing the terminology that is used with the Welsh grey literature of Wales (or vice-versa); and/or a lack of representation of Wales-specific social prescribing-related terminology within the peer-reviewed literature of terminology; and/or a failure of the peer-reviewed literature to accurately reflect the terminology that is used by the social prescribing workforce.

## Limitations

The present research possesses several limitations. These are discussed below.

The scoping review was conducted on a subject that is constantly evolving and was intended as a first step in the production of a glossary of terms for social prescribing (Newstead *et al.*, 2023), which itself is anticipated to be a first step in the identification and clarification of the terminology associated with social prescribing. Future iterations of the glossary will likely be needed as the landscape of social prescribing evolves, and these will require additional research to capture changes and additions to the language associated with social prescribing. However, given the period between conducting the searches for the scoping review and publication, new terms may have appeared in the social prescribing literature. We therefore supplemented our scoping review with a review of social prescribing literature produced within the last two years. Using the same inclusion/exclusion as described for the scoping review, we identified 329 potentially relevant documents and examined the full text of 68 documents (references for examined documents can be obtained at shorturl.at/jNY79). Perhaps reassuringly, we identified just eight terms that were not previously captured in the scoping review: link worker pathway (Sandhu *et al.*, 2022, link worker social prescribing intervention (Griffiths *et al.*, 2023; Wildman & Wildman, 2023), therapeutic community gardening (Wood *et al.*, 2022), green exercise initiatives (Massey *et al.*, 2023), green poverty (Gerodetti & Foster, 2023), and animal-assisted activities (Howarth *et al.*, 2021).

The terms used within the searches conducted for the scoping review were identified by members of the WSPRN and the WSSPR steering group, which contains a diversity of professionals that have contact with and represent a broad spectrum of social prescribing groups across Wales. While we are therefore confident that we identified at the very least, the majority of the most useful terms for the searches, we acknowledge that the decision to not contact external groups directly may mean that there are terms that we missed.

We acknowledge the limitations of the data from this research and that the inferences made from these data may not accurately reflect the representation or use of terminology by the workforce who encounter and deliver social prescribing, within Wales and beyond. It is likely that some terms used in everyday practice have evaded capture by the literature and by this scoping process.

## Conclusion

This research, to the best of our knowledge, is the first to attempt to identify, collate, and categorise the terminology associated with social prescribing and serves to highlight the diversity of terminology associated with social prescribing and the lack of standardisation of terms that describe essential components of the social prescribing process. Terminology differs not only across nations of the UK but also across sectors. Such diverse and confusing terminology can hinder comprehension of what social prescribing is and what the pathway involves, as well as impair effective communication between professionals and with the general public (Husk *et al.*, 2016; The King’s Fund, 2020; Tierney *et al.*, 2020; Wallace *et al.*, 2021).

The research indicates a bias of terminology originating from articles that examine research/interventions in England and that are authored from the perspective of health or health and social care. It is beyond the scope of this research to ascertain whether or not this terminology accurately reflects the language used by the workforce who encounter or deliver social prescribing. However, the research indicates that there are nation- and sector-specific terms that may not be adequately represented in the literature at large. Looking forward, it will be important to not only work towards standardising the terminology associated with social prescribing, across nations, and sectors of the UK but also to ensure that there is adequate representation of culturally relevant social prescribing terminology within the literature. To this end, more needs to be done to ensure that CVSOs, and the nations of the UK within which they reside, are able to report on the efficacy of their interventions within the peer-reviewed literature. Additionally, it will be important to ensure that the terms included in the literature do not solely come from literature sources but are also obtained from the workforce who encounter and deliver social prescribing.

The findings from this review represent the first step towards the development of an evidence-based glossary of terms for social prescribing and have laid the foundation for additional research. The development of the glossary of terms (Newstead *et al.*, 2023) has already been incorporated into the Welsh Government’s National Framework for Social Prescribing (due for release in December 2023) and their consultation document (Welsh Government, 2022).
